# Editorial: A century of health professions' education, training, academic research and service at Makerere University, Kampala, Uganda

**DOI:** 10.4314/ahs.v22i2.2S

**Published:** 2022-08

**Authors:** Damalie Nakanjako, Francis Omaswa

**Affiliations:** 1 Department of Medicine, School of Medicine, Makerere University College of Health Sciences, P.O. Box 7072, Kampala, Uganda; 2 The African Centre for Global Health and Social Transformation (ACHEST), 13 B John Babiha (Acacia) Ave, Kampala, Uganda

The Makerere University College of Health Sciences traces its history to 1924, when Makerere University Medical School was established as a medical training college; as the first and the oldest medical training University unit in East and Central Africa; drawing trainees from Kenya, Tanzania, Uganda and smaller numbers from Malawi, Zambia and Zimbabwe. Subsequently it became a Faculty of Medicine, based at Mulago National Referral and Teaching Hospital complex. The early qualifications were Licentiates that were recognized by the General Medical Council of the UK. After Independence these qualifications were converted into the Bachelor of Medicine and Bachelor of Surgery of the University of East Africa until Makerere was established as an independent University with the Faculty of Medicine and other regional universities in Nairobi and Dar es Salaam had started medical training programs. The new Medical school in Nairobi was facilitated by cohorts of Makerere students and teachers locating to Nairobi for periods of time to prepare the Kenyatta National Hospital to handle medical students.

“*I was in one of the groups taken to Nairobi for one term to prepare the hospital to handle students during my second year*” said Professor Omaswa, Executive Director of the African Center for Global Health and Social Transformation (ACHEST), and former Director General of Health Services in the Ministry of Health in Uganda

The Faculty of Medicine expanded medical training to other health professionals including pharmacists, dental surgeons, nurses, radiographers, biomedical engineers, environmental scientists; among others, hence the transformation to a College of Health Sciences (MakCHS). MakCHS consists of the School of Medicine, School of Public Health, School of Biomedical Sciences and School of Health Sciences, and most recently the School of Dentistry has been established; and a School of Pharmacy is underway. MakCHS continues to lead health research and innovations at Makerere University and the region.

**MakCHS vision** is to be a thought leader of knowledge generation for societal transformation and development, with a mission to impact the national health system, through transformational teaching, research and innovation for societal development. The six core values are. innovativeness and adaptability to change, social accountability, diversity, excellence and quality, equity and social justice, and professionalism.

**Innovations in science and health care:** Scientists at MakCHS focus on solving the most pressing health problems of the people of Uganda, and the sub-Saharan Africa region. As early as 1948, scientists at Makerere University described endomyocardiofibrosis, a new obstructive disease of heart muscle among Ugandan children^1^, ^2^, and its pathology and treatment were subsequently studied and implemented^1^, ^3^–^7^. Similarly, the Buruli ulcer, caused by Mycobacterium ulcerans infection, was described for the first time in Kinyara, Uganda, and subsequendy in Madi district, an area along the river Nile in the north of the country^7^–^9^. Burkitt's lymphoma was first documented in Uganda, initially described as a sarcoma of the jaw and subsequently, in 1962, shown to be a distinct form of Non-Hodgkin lymphoma^10^–^12^. This led to the establishment in 1967 of a dedicated cancer research institute, the Uganda Cancer Institute (UCI)^13^. Similarly, in 1970, research on Kaposi's sarcoma was conducted in Uganda by Dr. Kyalwazi through early immunological studies, where a striking impairment in the delayed hypersensitivity response to dinitrochlorobenzene was noted in patients with a “malignant” type of tumor^14^. Fast forward to 1985, when “Slim disease”, HIV, was described by three Makerere University scientists as a new disease associated with HTLV-III infection^15^. Subsequently, the 1999 landmark HIVNET 012 study showed that single-dose Nevirapine at the onset of labor and a single dose to the infant led to a 42 percent reduction in maternal to child HIV transmission, providing the developing world with a cheap and simple option to protect thousands of children born by HIV-infected mothers^16^, ^17^. Makerere also established the first cardiac catheterization laboratory in sub-Saharan Africa and provided closed cardiac surgery services to the region from the 1960s. From that time until a decade ago, all Heads of Department at the National Referral Hospital were Univeristy employees and they also doubled as the chief advisors to the Ministry of Health in their respective areas of specialization; this providing a seamless continuum of service, teaching and research as the three legs of the stable African stool.

These health innovations and research have continued, including several studies to improve the diagnosis and treatment of HIV and associated opportunistic infections in hospitals and communities^18^–^22^ and unique patterns of immunological recovery^23^–^25^. More recently, a KIR B centromeric gene (2DS5) present in Ugandan women was reported to be protective against pre-eclampsia; a leading cause of maternal mortality in Uganda^26^, ^27^. Consequently, several centers of excellence in healthcare training and research have emerged including, but not limited to, the Infectious Diseases Institute (IDI), Makerere University Walter Reed Project (MUWRP), Rakai Health Science Project (RHSP), Makerere University Case Western Reserve collaboration, Makerere University John Hopkins University (MUJHU) collaboration, the Makerere Lung Institute (MLI), Uganda Heart Institute (UHI) and the Makerere University Joint AIDS Program (MJAP); which are presented in this African Health Sciences supplement as Makerere celebrates 100 years of higher education.

Health scientists at MakCHS continue to provide invaluable support to national health priorities, including current contributions to the national response to the COVID-19 pandemic. MakCHS scientists constitute half of the national COVID-19 task force^28^, ^29^. The Schools of Medicine, Public health, Biomedical Sciences, and Health Sciences, and the Infectious Diseases Institute at Makerere University's College of Health Sciences have been at the forefront of COVID-19 innovations in diagnostics, case management, surveillance, modelling impact, and public health interventions against the spread of the pandemic^28^, ^30^, ^31^. The government of Uganda, through the Makerere Research and innovation fund (MakRIF) supported several MakCHS faculty who won competitive research awards to support multi-disciplinary approaches to control the spread of SARs-COV2 and the impact of the disease^31^.

**Innovations in medical education:** The college of health sciences continues to lead innovation in education with the adoption of problem-based learning, competence-based medical training and community-based education, research and service (COBERS) as integral parts of medical training to produce medical graduates that are leaders in societal transformation. MakCHS continues to be a part of global health professions training networks including the Association of Academic Health Centers International (AAHCI), the regional AAHCI-Eastern-Africa^32^, and the Consortium of Universities for Global Health (CUGH); among others. Common Goals of Academic Health Centers are a) structuring health professions education to meet changing and evolving societal needs, b) Linking research to improved health outcomes, and c) transforming patient care based on population needs and priorities^33^. Similarly in June 2022, MakCHS was competitively awarded to host the Eastern Africa regional institute of the Foundation for the Advancement of International Medical Education and Research (FAIMER). The Eastern Africa FAIMER Regional Institute (EAFRI), hosted by MakCHS in Kampala, Uganda, will utilize a multilateral partnership model, with Mbarara University of Science and Technology (MUST), Faculty of Medicine in Mbarara, Uganda serving as MakCHS' regional partner. This multilateral partnership model will expand FAIMER's reach to promote health professions education and workforce development in additional areas of interest. Makerere was a member of the Medical Education Partnership Initiative (MEPI) and contributed significantly to the establishment of the African Forum for Education and Research in Health (AFREhealth). In Uganda, Makerere graduates have provided leadership to the many new medical training programs in the country. *“My vision is to foster an environment that develops life-long learners and transformational leaders in science and innovation to meet our community's health needs in the 21^st^ Century”,* said Dama-lie Nakanjako, a Professor of Medicine and Principal for MakCHS.

**Training leaders for the next generation:** In addition to serving as researchers and scientists, medical doctors trained at Makerere University have contributed to Uganda, East Africa, Africa and globally in technical and political leadership roles. These leaders include: the late Dr. Samson Kisekka, who served as prime minister (1986 - 1991) and vice president of Uganda (1991 – 1994); Stephen Malinga, the former minister for disaster relief and refugees in the Ugandan cabinet (2011 – 2013); Ruhakana Rugunda, Prime minister (2014–2021); Cris-pus Kiyonga, former minister of defense in the Ugandan cabinet (2006–2016); Specioza Kazibwe, a surgeon, women's activist, and former vice president of Uganda (1994–2003); Gilbert Bukenya, a former vice president of Uganda (2003 – 2011); Christine Ondoa, former minister of health (2011 – 2013) and director of the Uganda AIDS Commission since 2014; Maggie Kigozi, the former executive director of the Uganda Investment Authority; and the current minister of health, Dr Jane Ruth Achieng^34^ and many other influential MakCHS alumni. In Kenya and Tanzania many ministers, directors of medical services and academics are graduates of Makerere Medical school. There are also many who have served in the World Health Organization (WHO) and other UN agencies at different levels and many have won accolades and awards from round the world.

*“It is with great pleasure and enthusiasm that I serve as a clinician, scientist, trainer, and mentor to lead a team of talented Ugandans who have devoted their service to maintaining the highest quality of training, research, healthcare, and exemplary professional leadership at Makerere University's medical school”* said Damalie Nakanjako, a Professor of Medicine and Principal for MakCHS.

**Transforming the health workforce:** Uganda aspires to optimize more national gains from her well-trained Health Work Force (HWF) through strategic interventions to prevent brain drain as many researchers and clinicians are lost to ‘greener pastures’ due to current limited absorption capabilities of the national HWF pool^35^. In the past few decades there has been a call for transformation of health professions training from all over the globe. Uganda has participated actively in several reforms and interventions to improve the HWF, one of the six health systems building blocks, both in quality and numbers. It has been projected that there will be shortfall of 18 million health workers by 2030, mostly in low- and lower-middle income countries including Uganda. By 10th June 2022 Uganda had only 8453 registered medical doctors and 415 dentists in Uganda; the numbers grow smaller with further categorization to the different specialties and sub-specialties. This is partly due to chronic under-invest-ment in education and training of health workers, under absorption of the trained health workers, as well as weak human resources for health planning, management and information systems; among other factors. This situation has been made worse by the COVID -19 pandemic.

This is therefore a call to action for inter-ministerial collaboration (Ministry of Education, Ministry of Health, Ministry of Finance, Ministry of Gender, Labor and So-cial transformation and others) to work with Universities, Health Professional Associations and educators in Uganda, to ensure the provision of adequate, accessible, acceptable and quality health workforce for the people of Uganda, and the globalized world. The gains from the last century of health professions education have set a foundation for all stakeholders to utilize to build a multi-disciplinary strategy to provide the health workforce required for healthy and wealthy nation(s) for the next 100 years.
